# Detecting expertise in decision making under pressure: a virtual reality assessment environment and empirical evaluation

**DOI:** 10.1186/s41235-025-00695-6

**Published:** 2026-01-08

**Authors:** Matthew B. Thompson, Varun Gandhi, Alexandra Richardson-Newton, Guillermo Campitelli

**Affiliations:** 1 School of Psychology, Murdoch University, Murdoch, Australia; 2 Centre for Biosecurity and One Health, Harry Butler Institute, Murdoch University, Murdoch, Australia

**Keywords:** Decision-making, Expertise, Human perception and performance, Individual differences, Virtual reality

## Abstract

Professions such as military, aviation, submarine operation, and emergency response require individuals to navigate complex environments characterized by limited information, stringent time constraints, and significant pressures. Effective decision making under pressure is crucial in safety–critical professions, yet measuring this expertise remains challenging. Inspired by the military context, this article introduces the virtual reality decision-making expertise (VR-DMX) environment, designed to evaluate decision-making expertise under time constraints within a virtual reality scenario. VR-DMX simulates an amusement arcade where users must decide how to allocate time across various games to maximize ticket earnings. Through two validation studies (*N* = 60 and *N* = 76), we examined two metrics: Total Tickets (measuring overall performance) and DMX score (isolating decision-making quality). Both metrics demonstrated symmetrical distributions without floor or ceiling effects, with coefficients of variation comparable to established individual difference measures (32.4–37.4% for Total Tickets; 20.8–27.6% for DMX score). The moderate correlation between metrics (meta-analysis *r* = 0.771, 95% CI [0.599, 0.943]) indicates they measure related but distinct constructs. Our findings indicate that VR-DMX effectively differentiates individual performance levels and captures a distinct decision-making component that is separate from general cognitive abilities. Comparing decision-making expertise between professionals in safety–critical fields with those without such experience would be a sensible next step to help validate the potential for selection and training applications. VR-DMX was designed to measure decision-making expertise in safety–critical contexts, and initial validation data demonstrating effective differentiation of individual performance levels suggest that continued development could fulfill this design intention for applications in selection, training, and performance prediction.

**Supplementary Information:**

The online version contains supplementary material available at 10.1186/s41235-025-00695-6.

## Significance Statement

In many safety-critical professions—such as aviation, submarine operation, firefighting, and emergency response—effective decision-making under pressure can mean the difference between safety and disaster. Individuals in these roles must routinely make rapid high-stakes choices in stressful, unpredictable environments; yet accurately measuring and understanding the decision-making capabilities that facilitate optimal performance under these conditions remains challenging. To address this real-world need, we developed the Virtual Reality Decision-Making Expertise (VR-DMX) environment, a novel virtual reality assessment designed to measure an individual's ability to make effective decisions when facing time constraints. VR-DMX simulates a realistic, fast-paced scenario—an amusement arcade—where users strategically allocate their limited time across tasks to maximize reward. Validation testing indicates that VR-DMX reliably detects individual differences in decision-making expertise, demonstrating its ability to discriminate among varying levels of performance and decision quality clearly. Because VR-DMX appears to capture variations in the ability to allocate attention and prioritize tasks strategically under stress, initial validation data suggest that continued development could enable applications in training and selection within safety-critical domains, potentially guiding practical improvements in training and selection processes to enhance operational safety and effectiveness in sectors where poor decisions can have severe consequences.

**Supplementary Information:**

The online version contains supplementary material available at 10.1186/s41235-025-00695-6.

## Introduction

In safety–critical professions, effective decision making is pivotal, often carrying life-or-death implications. Pilots, submariners, and emergency responders are among those who routinely operate in environments that are inherently complex and characterized by constraints such as limited information, acute time pressures, and high stakes. This article introduces a virtual reality test environment designed to measure decision-making expertise under time pressure, and presents two studies exploring its relationship with other measures of individual differences in decision making. The Virtual Reality Decision-Making Expertise (VR-DMX) contains a virtual reality scenario of an amusement arcade with multiple rooms and machines with arcade games. Users must make decisions about how much time they spend in each game to maximize the number of tickets gained. This environment is designed to assess decision-making expertise in safety–critical professions, such as military, aviation, submarine operation, and emergency response.

We first briefly discuss the characteristics of extant decision-making environments in two main lines of research in decision making; second, we discuss the need for decision-making expertise measures in safety–critical professions; third, we introduce VR-DMX; fourth, we present two studies that explore the measure’s distributional characteristics and its correlation with other measures of individual differences. Finally, we synthesize the findings from both studies.

### Decision-making measures

Expert decision making refers to the ability to make fast, accurate, and adaptive decisions in complex, uncertain, and time-pressured environments, typically informed by extensive domain-specific experience and intuitive pattern recognition. Two main lines of research have been followed in expert decision making: (1) highly controlled laboratory studies (see Campitelli & Gobet, 2010, for an overview of this approach), and (2) highly uncontrolled naturalistic decision making in the field (Klein, 2008). Both lines of research uncovered the importance of domain-specific pattern recognition when making decisions in suboptimal environments. To identify measures aligning with our purpose, we explored two repositories of existing decision-making measures, one tied to the first approach and the other to the second. The first repository is the Decision-Making Individual Differences Inventory (Appelt et al., 2011), a free online repository containing a catalog of over 200 measures of individual differences used in the decision-making literature (https://sjdm.org/dmidi/). The second repository is maintained by the Naturalistic Decision-Making Association and contains a catalog of more than 40 measures used in the naturalistic decision-making research community (https://naturalisticdecisionmaking.org/new-ndm-tools/).

The Decision-Making Individual Differences Inventory categorizes the measures into seven groups: decision making, risk attitude, cognitive ability, motivation, personality inventories, personality constructs, and other measures. As our focus lies on decision-making expertise, only the decision-making measures are relevant for our purpose. The most pertinent measures within the inventory are those assessing decision competence, including the Adult—Decision-Making Competence (ADMC) measure (Bruine de Bruin et al., 2007), the Older Adult—Decision-Making Competence (OADMC) measure (Finucane & Guillion, 2010), the Youth—Decision-Making Competence (Parker & Fischhoff, 2005), Decision Outcome Inventory (DOI; Bruine de Bruin et al., 2007), the Pre-Adolescent Decision-Making Competence measure (Weller et al., 2012), and the Problem Solving Inventory (PSI; Heppner, 1988).

Among these, only the various versions of the Decision-Making Competence measure serve as performance measures. In contrast, the Decision Outcome Inventory requires test-takers to indicate the decisions they make in life, but not in suboptimal situations, which is the focus of our approach. Additionally, despite its name, the Problem Solving Inventory is not a performance measure. Therefore, only the different Decision-Making Competence measures are relevant to our inquiry. While these measures are highly valuable, they assess typical decision-making skills such as resistance to framing, overconfidence, and understanding probabilities and rules in formal mathematical/logical models. These decision-making tasks gained prominence through the work of Kahneman and Tversky (e.g., Kahneman & Tversky, 1984; Tversky & Kahneman, 1974). However, these tasks do not measure decision making in suboptimal situations or under time pressure, which is typically the case in safety–critical professions.

The repository maintained by the Naturalistic Decision-Making Association is more relevant for the purposes of this article as decision making in suboptimal situations and safety–critical professions is the focus of the naturalistic decision-making community, with the work of Gary Klein (e.g., Klein, 2008) being foundational in this field. The tools in this repository are classified into nine categories: knowledge elicitation, cognitive specifications and representation, training, design, evaluation and assessment, teamwork, risk assessment, measurement, and conceptual descriptions. The three categories relevant to our purposes are training, evaluation and assessment, and measurement. Among the training tools, The ShadowBox (e.g., Klein & Borders, 2016; Klein et al., 2013) is relevant for our purposes. However, tools in the Evaluation and Assessment category are not measurement instruments; instead, they offer frameworks or approaches for assessments. In the Measurement category, Klein (2008) provides guidelines for measuring macrocognitive functions, not a measurement tool, while Hoffman et al. (2010) introduce a statistic to evaluate the learnability and resilience of tools, not the decision-making expertise of people.

### Rationale for the virtual reality decision-making expertise (VR-DMX) environment

Although the two repositories display valuable tools, none are suitable for measuring decision-making expertise under time pressure while maintaining a general applicability across multiple domains or professions. For this reason, we decided to create a new tool: VR-DMX. VR-DMX is a fully immersive virtual reality environment for detecting individual differences in decision-making ability in a realistic setting. This approach has the advantage of being laboratory-based (hence, gaining control over variables; see Thompson & Tangen, 2014) and being contextualized (i.e., a virtual reality realistic scenario), which allows us to measure decision-making skill in a realistic situation. By adopting this approach, we will be able to investigate an essential component of decision making under suboptimal conditions: deciding how much time or resources to spend in different situations or tasks.

We aim to capture the essence of decision making under challenging circumstances while ultimately providing a reliable assessment that can inform the selection, training, and performance prediction of professionals in such demanding fields. Our approach involves crafting an environment that strikes a balance between specificity and generality, a hybrid that can be applied across various professions while maintaining focus on the nuances of decision making in suboptimal situations. The environment, therefore, seeks to remain sensitive to the unique challenges inherent to various safety–critical roles and to preserve a level of generalizability that allows insights from one domain to cross-pollinate with others.

We identified three main challenges: (1) maintaining a balance between predictability and specificity, ensuring the environment remains relevant across various domains without compromising its internal predictive accuracy; (2) achieving suitable ecological validity while exercising experimental control, ensuring the environment represents aspects of real-world situations; and (3) differentiating between general expertise and decision-making acumen, ensuring that the environment measures an individual’s decision-making capability rather than their domain-specific knowledge.

VR-DMX aims to identify a common element of decision-making aptitude that spans various professions, thus ensuring both high predictability and broad applicability. This involves a scenario in which individuals allocate time among competing tasks without clear indications of their relative importance or profitability, akin to players in a game arcade seeking to maximize ticket winnings. Importantly, the specific arcade games themselves are not the primary focus. Rather, our interest lies in how individuals decide whether to continue playing a game or shift to another. However, we selected games that incorporate traditional psychological tasks, as a secondary aim of VR-DMX is to assess psychological abilities individually.

Chess has served as a model for understanding decision-making since the foundational work of Herbert Simon (e.g., Chase & Simon, 1973; see Campitelli & Gobet, 2010, for Herbert Simon's expertise approach to decision making) and has been adopted by naturalistic decision-making researchers (Calderwood et al., 1988; Klein et al., 1995). We use chess to provide an example of the differences between domain-specific expertise and metacognitive processes that are less domain-specific yet critically affect performance. Like the VR-DMX environment, chess is played under time control, requiring players to manage how they distribute their thinking time across each move they make. Expertise in chess is primarily explained by the learning and storage of domain-specific knowledge structures in long-term memory (i.e., configurations of chess pieces on a chess board), such as chunks (e.g., Chase & Simon, 1973), templates (e.g., Gobet & Simon, 1996), and retrieval structures (e.g., Ericsson & Kintsch, 1995).

However, it is well-known that some players distribute their thinking time very poorly, spending excessive time deliberating on moves in non-decisive positions (see Chacoma & Billoni, 2025, for a machine learning measure of move decisiveness) where any "good enough" move would be appropriate. This poor resource allocation leads these players to have very little time to think in positions with high decisiveness (i.e., positions in which finding the best move is complex and failing to find the best move would lead to a disadvantageous position), resulting in serious mistakes. Hence, their poor decision-making capability prevents them from achieving their full potential (see Campitelli et al., 2014, for a mathematical model of expertise that differentiates domain-specific knowledge from actual performance). Similarly, in the VR-DMX environment, participants must apply their emerging knowledge of individual games while monitoring their performance, learning about the profitability of each game they explore, and deciding whether to continue playing a game or switch to another alternative. This parallel illustrates how strategic resource allocation represents a distinct component of decision-making expertise that could be measured independently of domain-specific knowledge.

The VR-DMX task is fundamentally designed to engage domain-general cognitive processes that we hypothesize are central to decision-making expertise in safety–critical contexts, deliberately abstracting away from domain-specific knowledge to focus on transferable cognitive capabilities. This approach reflects the theoretical distinction between fluid cognitive processes that transfer across domains and crystallized context-specific knowledge. While domain experts possess superior knowledge structures within their specialized areas, the underlying cognitive processes of strategic thinking, resource allocation, and adaptive decision making represent more general capabilities that can be applied across diverse contexts. Our task design intentionally minimizes prior knowledge requirements while maximizing engagement of these transferable processes through novel game contexts that require strategic learning rather than recall of established procedures.

### Description of the VR-DMX

**Fig. 1:**
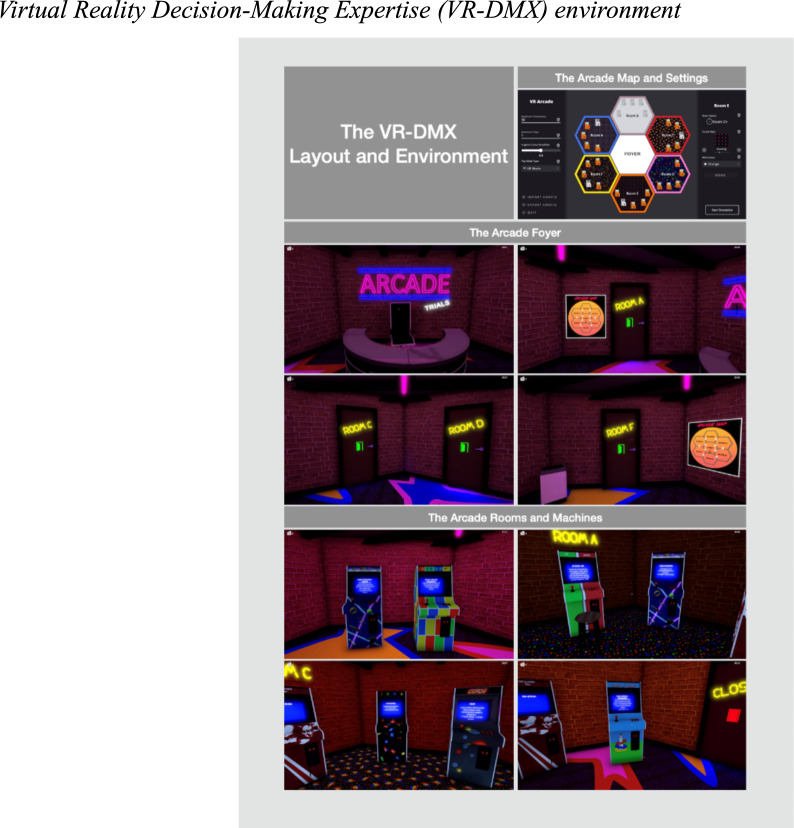
Virtual reality decision-making expertise (VR-DMX) environment. Note. The top right panel shows a graphic depiction of the virtual reality environment, which contains a foyer (four middle panels) and 5 active game rooms (example of one room in the bottom four panels), which can be accessed by their corresponding door. Each room contains 4 or 5 games. In the version used in the studies reported in this article, there were twenty-one machines, each with one arcade game

When participants begin, they are in the lobby of an amusement arcade, and they can choose among five rooms (see Fig. 1). Players use their controller to navigate the arcade and enter different rooms. Each room contains five arcade machines, each offering a different game. Once inside, participants can approach and choose which machine to play. If the machine is active, it will display the name of the game, a brief description, and the number of tickets players can win. Arcade machines that are not hosting a game show an "out of order" sign. In each game, the participants acquire tickets according to how well they perform the task at hand.

**Fig. 2:**
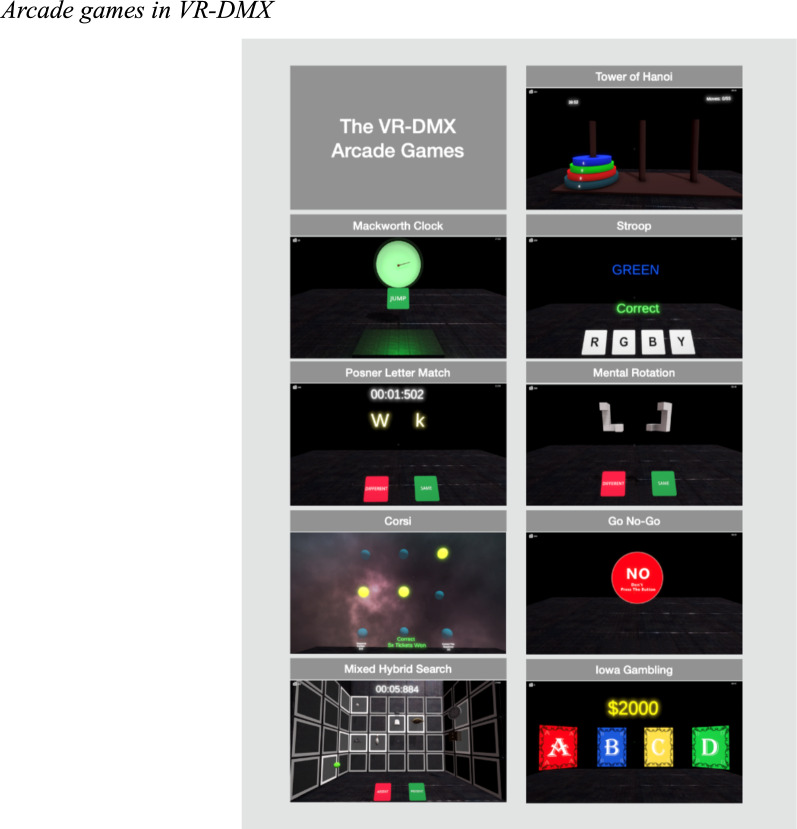
Arcade games in VR-DMX. Note. The panels show nine of the games the participants played in VR-DMX. The total number of psychological tasks was 9, for which we created several versions of each task, totaling 21 arcade games

The VR-DMX encompasses psychological tasks, such as the Corsi block-tapping test, the Go No-Go, the Iowa Gambling task, the Mackworth Clock, the Tower of Hanoi, the Stroop test, the Mental Rotation task, and the Posner Letter Match task. Each task is adapted for a virtual reality context, maintaining the integrity of the original tests while also providing a novel medium for participant engagement. The virtual reality adaptations of each task are presented in Fig. 2 and Appendix A shows a brief description of each of the psychological tasks.

Critically, participants in the VR-DMX Arcade exercise autonomy in managing their time and efforts within this simulated environment. They are given the agency to strategize and make choices about which games to engage with, based on the potential ticket rewards displayed. A player might be enticed to try a game that advertises a high reward, but if they perceive a discrepancy between their efforts and the tickets earned, they have the freedom to exit the game prematurely and explore other options. Participants are also able to move freely between different tasks, a process that involves both physical navigation within the virtual space and temporal management, as switching games consumes valuable time. Remembering the locations of the various games can be challenging, and the layout of the arcade adds another layer to the strategic planning required for maximizing performance.

While VR-DMX does not impose the acute, deadline-driven time pressure often associated with safety–critical decision-making scenarios, our task captures a different but equally important form of time pressure that characterizes many real-world decision-making contexts: sustained resource allocation under temporal constraints and uncertainty. Participants face a continuous need to decide whether to remain in their current game or switch to another alternative, creating implicit time pressure through moment-by-moment opportunity cost decisions rather than explicit countdown timers. Every second spent in one activity represents foregone opportunities in alternatives, requiring participants to continuously evaluate whether their current time allocation remains optimal and creating ongoing cognitive load and decision stress.

This form of temporal constraint is partly representative of many safety–critical contexts where professionals must optimize resource allocation over extended periods rather than make split-second responses. Submarine operations during extended missions require crew members to continuously allocate attention and effort across multiple systems and priorities, while emergency coordinators must manage resources across multiple ongoing incidents. These scenarios involve sustained decision making under time constraints and uncertainty, where pressure arises not from immediate deadlines but from the continuous need to optimize limited resources across competing demands. The cognitive processes engaged by sustained time pressure differ meaningfully from those involved in acute deadline pressure, yet both are relevant to safety–critical performance. Our design aims to engage executive control processes for managing competing priorities, working memory for tracking multiple ongoing commitments, and metacognitive monitoring for evaluating allocation strategy effectiveness.

### Measure of expertise 1: total tickets

We developed a measure of performance in the task (i.e., overall expertise) and a measure of the quality of the choices made (i.e., decision-making expertise). The measure of overall expertise is the total tickets earned in the arcade experience, where a player who has earned more tickets is deemed to have performed better. The measure of decision-making expertise is called DMX score, by which the higher the DMX score, the higher their decision-making expertise. Decision-making expertise refers to a person’s ability to make good choices in a complex environment, specifically selecting options that lead to better outcomes over time. In the context of the VR-DMX, this refers to participants’ ability to choose the most optimal games to play in order to maximize ticket earnings within the time limit. By design, this decision-making expertise measure subtracts out the influence of each participant’s skill level on the games themselves.

The Total Tickets measure quantifies participant performance (i.e., overall expertise) in the VR-DMX. Overall expertise is measured by the number of tickets earned by a player in the arcade within the limited time provided (i.e., 30 min). Better performance is indicated by a greater number of tickets earned and poorer performance is indicated by fewer tickets earned. Participants earn tickets based on their performance in each game that they play. Therefore, Total Tickets can be calculated by summing the tickets earned across all plays.

It is important to note that Total Tickets earned is a function of performance in each game, and choosing the most profitable games. A participant with high expertise in a game will naturally perform better and earn more tickets than a participant with low expertise. For example, a participant with greater visual working memory will perform better in Corsi, compared to a participant with poorer visual working memory, as the task requires accurately remembering and reproducing spatial sequences. Alternatively, a participant who has better game selection ability will earn more tickets than a participant with poorer game selection ability. As such, a participant who chooses more profitable tasks to play will also naturally earn more tickets than a participant who chooses less profitable tasks.

Given that Total Tickets is influenced by game performance and game selection, this measure fails to tell us whether a participant’s overall level of performance is attributable to their expertise with the individual tasks or to their task selection ability. In fact, it is entirely possible for two participants to have the same level of overall expertise (e.g. Total Tickets_Participant A_ = Total Tickets_Participant B_ = 1000), but have different levels of task performance and task selection. For example, Participant A may perform poorly on the tasks but may compensate for this by choosing more profitable tasks. In contrast, Participant B may perform very well on the tasks but may be hampered due to their selection of less profitable tasks. As such, although we expect that the Total Tickets measure will still predict important individual differences between people in the decision-making component of the measure, it is ‘contaminated’ by the dual influences of task performance and task selection.

Self-evaluation and metacognitive monitoring are likely core cognitive processes operating within VR-DMX, functioning at multiple temporal and strategic levels. Participants must engage in continuous metacognitive monitoring of their current performance while simultaneously evaluating the profitability of their time allocation decisions. This creates a kind of dual cognitive load where individuals must both execute specific game tasks and make higher-order strategic decisions about resource allocation under temporal constraints. The VR-DMX incorporates structural limitations that prevent simple exploitation strategies from dominating performance. For instance, a game like Mental Rotation may allow only two plays maximum, while others have diminishing ticket yields over time. These constraints ensure that good performance requires strategic switching decisions rather than mere persistence in high-ability domains. The task design rewards rational persistence when justified by good performance, but only when participants have accurately evaluated game profitability through initial exploration.

The cognitive processes underlying effective VR-DMX performance may parallel those required in safety–critical professions. Consider a submariner responding to an emergency who must evaluate whether to continue current repair efforts or switch to addressing a different critical system. Like our arcade scenario, this requires continuous self-monitoring of task effectiveness, strategic evaluation of alternative courses of action, and resource allocation decisions under time pressure. The submariner cannot simply persist with familiar procedures but must dynamically evaluate whether current approaches remain optimal given changing circumstances.

### Measure of expertise 2: DMX score

To mathematically distinguish between game performance (i.e., game expertise) and task selection (i.e., decision-making expertise), we needed to translate our conceptual definition of decision-making expertise into an operational one. That is, what does it mean for someone to make a ‘high quality decision’ in the VR-DMX? To answer this, we reflected on what ‘quality decisions’ look like in safety–critical professions and domains of expertise. For instance, consider a submariner responding to a time-sensitive emergency where they need to decide between high reward tasks (e.g., extinguishing a fire) or low reward tasks (e.g., checking weather conditions). Supposedly, this type of decision making requires an understanding of the ‘reward’ gained for completing different tasks and an understanding of the time required for each of these tasks. Additionally, ‘reward’ may be dynamically changing (i.e., checking weather conditions may be ‘low reward’ during an on-board fire emergency but may be ‘high reward’ during an impending storm) and is aligned to some overall outcome (e.g., safety—extinguishing a fire increases safety during a fire emergency, but checking the weather probably would not).

Our conceptualization of decision-making expertise is one that reflects this ability to discriminate between high and low reward tasks. A person who has decision-making expertise will more frequently select high reward tasks, while a person without decision-making expertise will select low reward tasks. Given that the overall outcome of the VR-DMX scenario is to earn as many tickets as possible within the limited time available, ‘reward’ is operationalized as the number of tickets earned per second by a participant (earlier referred to as ‘profitability’ of a task). A participant who has high decision-making expertise will more frequently select highly profitable games, while a participant with low decision-making expertise will select games that are low in profitability (i.e., those do not provide many points per second).

Operationally, this is quantified by assigning a value to each decision made by the participant. We considered that the participant makes a decision every second, which is to keep playing the game that they are playing or to move to another game. Given that the game lasts 30 min, we assume the participant makes 1800 decisions. The value of the decision is determined by the profitability value of the game chosen (i.e., the current game or the new game) at the time the choice is made. This is important because the profitability value of each game changes as the participant plays a game and learns the value of the game they played. We then add the values of the 1800 decisions to obtain the final DMX score.

Determining the profitability value of each game at each second of the 30 min session is a complex endeavor. Although we explain our approach briefly below, we also refer interested readers to the Supplementary Materials for a detailed description of the computational steps taken. The difficulty in assigning profitability values to each task is that we want to capture the information available for each participant at the time they make each decision. At the beginning of the test, all the games have the same profitability value because the participant does not have information on the profitability of each game. That information is obtained by playing the game and observing the number of tickets gained in the game as a function of the time spent playing the game. After playing the first game, that game has a value for the participant and all the other games have an uncertain value, which will change after playing additional games.

The profitability of each game is determined at each second by indicating the value of each game relative to the value of all the other games. In order to achieve that, we need to apply a normalization by which the sum of the values of all the games is 1, which requires the following steps. First, for the games played by the participant, the profitability of the game is obtained by calculating the number of tickets gained per second at a specific point in time. For example, if by time 340 s, the participant played Tower of Hanoi and obtained 0.06 tickets per second, the Tower of Hanoi has a profitability value of 0.06 tickets per second. To the other games, we assign the average profitability value of the games played. Here we make the assumption that the participant can only infer that, on average, the other games will have the same difficulty as the one played. Because at this stage, we only have the profitability value of one game, the average is the value of that game; hence, the value of all the other games is also 0.06. Here we have two options for normalization: We can add all the values assigned to each of the 21 games (in this case 0.06 × 21 = 1.26) and then divide the profitability of each game by 1.26 (i.e., 0.06/1.26 = 0.047). As we will show below, this normalization has a problem. Thus, we used a second normalization, which is to assign the rank of each option, and when there is a tie, the rank assigned is the average of the two corresponding ranks (e.g., if two options have a value of 0.02 and they are in the 12th and 13th rank, respectively, they both receive the rank 12.5). In this case, all the games have the same value; therefore, they all receive the same rank, which is the sum of all ranks from 1 to 21, divided by 21 (i.e., 11). In this case, both normalization methods are equivalent, but when the difference between the profitability of the most profitable game and the other games is large, the unplayed games will receive a very low value, indicating that the only logical decision for the participant is to keep playing the same game. For example, if by time 1025 s of the test, a participant played 8 games, with the following profitability values: game 1 = 0.024, game 2 = 0.050, game 3 = 0.070, game 4 = 0.001, game 5 = 0.003, game 6 = 0.030, game 7 = 0.082, game 8 = 0.010, the sum is 0.270, and the average is 0.03375; thus, the normalized profitability assigned to games 9 to 21, which were not played, is 0.03375. We then assigned the rank to each game: game 7 = rank 1, game 3 = rank 2, game 2 = rank 3, games 9 to 21 = [tie rank 4 to 16 = rank 10], game 6 = rank 17, game 1 = rank 18, game 8 = rank 19, game 5 = rank 20, game 4 = rank 21.

The mathematical formulation of our DMX score specifically evaluates decision quality based on observed profitability rather than absolute performance levels. The DMX score calculation treats each second as a discrete decision point where participants choose to maintain their current strategy or switch alternatives. This operationalization allows us to quantify decision quality independently of individual game expertise, addressing the fundamental challenge of measuring strategic thinking separate from domain-specific knowledge. Further, the metacognitive demands of VR-DMX extend beyond simple performance monitoring to encompass strategic planning under uncertainty. Participants must develop mental models of game difficulty and profitability based on limited sampling, then continuously update these models as they acquire new information. This process requires not only self-evaluation of current performance but also prospective evaluation of alternative strategies and their potential outcomes.

We now describe two studies we conducted to explore the statistical properties of the VR-DMX. Any measure that aims to measure expertise needs to show variability, so we investigated whether the DMX score and total tickets possess this characteristic. Moreover, we performed exploratory correlations with some measures that were used in decision-making research.

## Study 1

**Fig. 3:**
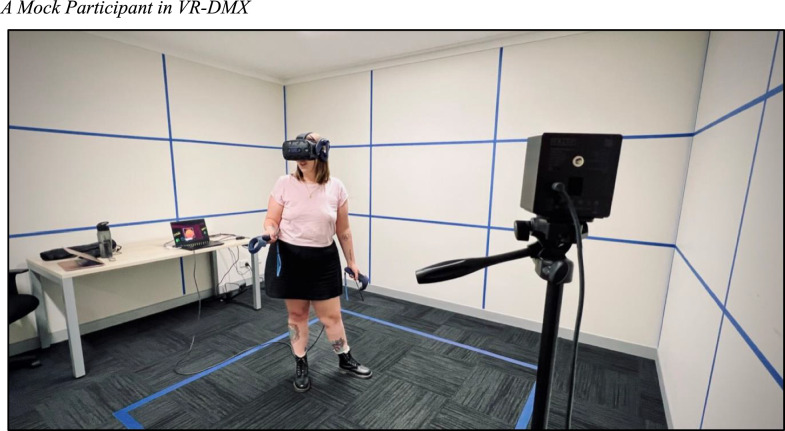
A Mock Participant in VR-DMX

The primary objective of Study 1 was to examine the distributional properties of two metrics: performance (Tickets) and Decision-Making Expertise (DMX) obtained while participants played in VR-DMX for 30 min (Figure 3 shows a mock participant playing VR-DMX). For a scale to detect individual differences effectively, it should allow for a wide range of possible values, with participants achieving scores spanning a broad spectrum. To this end, we evaluated the coefficient of variation. Additionally, we investigated the symmetry of the distribution through the assessment of skewness and kurtosis. A further objective was to measure the correlation between the new VR-DMX metrics and established cognitive style markers, namely cognitive reflection, need for cognition, and actively open-minded thinking', and alongside age. Participants were presented with the task of maximizing their performance (i.e., obtaining the maximum number of tickets they could) across several challenging cognitive tasks. To quantify performance in the VR-DMX, two distinct measures were formulated: Total tickets and DMX score, which were explained in the introduction. We also reported the number of unique games played (Games) and the number of switches among games (Switches).

To illustrate the DMX score calculation intuitively: Each second represents a decision point where participants either continue with their current game or switch to another. At every second, DMX assigns value based on the profitability (tickets per second) of whichever game the participant is currently playing, relative to all available options. Critically, profitability is determined by what the participant has learned through play up to that moment. Initially, all games have equal expected value, but as participants sample different games, they gain information about which are more rewarding. Higher DMX scores indicate consistently allocating time to games that appear most profitable given current knowledge, while lower scores reflect spending time in less profitable games when better alternatives have been discovered. For example, a participant who discovers a highly profitable game and continues playing it would earn a high DMX score, while a participant who persists in a low-yield game despite having sampled better alternatives would earn a lower score. This design isolates strategic decision-making quality—the ability to learn from experience and allocate time wisely—from individual game performance abilities.

### Method

#### Participants

Sixty-nine participants took part in the study. Seven participants were excluded due to technical issues (i.e., sessions ending midway due to software crashes or data failing to save), one participant was excluded as they were under the age of 18, and one participant was excluded as they withdrew due to illness. In total, valid data were collected from 60 participants (44 female, 15 male, 1 non-binary) with a mean age of 22.72 (*SD* = 5.48). Participants were predominantly university students or university-affiliated individuals. Recruitment occurred via two methods: Psychology students were recruited through the university’s research participation pool in exchange for course credit (54 participants), and additional participants were recruited via printed posters displayed across campus and were compensated $20 for their participation (6 participants). We initially aimed to detect medium-sized population effects (i.e., *r* =.30 or greater). A priori power analysis conducted using G*Power (*α* =.05, one-tailed; 1 − *β* =.80) indicated that a sample size of 67 participants was required. Due to time constraints, data collection concluded at 60 valid participants, which still afforded adequate power to detect effects of *r* ≥.32.

#### Materials

Participants used a HTC VIVE Pro 2 virtual reality headset to go through the Virtual Arcade (See Fig. 3). They also used a controller to virtually transport to different places of the arcade, to make choices, and to select objects. The Virtual Reality Arcade scenario used in this study has been extensively described in the Introduction. The participants also completed three cognitive scales: a 7-item version (Toplak et al., 2014) of the Cognitive Reflection Test (CRT; Frederick, 2005), an 11-item version (Baron et al., 2023) of the Actively Open-Minded Thinking Beliefs (AOMTB) scale (Stanovich & West, 1997), and the short version (18 items) of the Need for Cognition scale (NCS; Cacioppo et. al., 1984).

The 7-item version of the CRT contains seven problems. The common characteristic of these problems is that there is an alternative that seems to be the correct answer, but it is incorrect (see Campitelli & Gerrans, 2014 for a mathematical model of the CRT). Thus, participants need to inhibit providing that response and find the correct alternative, which is relatively easy. Given that the solution of the problem is relatively easy, Fredrick (2005) proposes that this test measures something different from mathematical ability: cognitive reflection (see also Campitelli & Labollita, 2010). The minimum score in this test is 0 (i.e., 0 correctly answered problems) and the maximum is 7 (i.e., 7 correctly answered problems). We used a multiple-choice version of the CRT, in which participants were given four options to choose from in each question (see Sirota & Juanchich, 2018 for a validation of this multiple-choice version of the CRT). The AOMTB scale assesses beliefs about whether actively open-minded thinking is good. It is based on the concept of actively open-minded thinking concept developed by Baron (1994). Statements such as “True experts are willing to admit to themselves and others that they are uncertain or that they do not know the answer” or “People should take into consideration evidence that goes against conclusions they favor” are presented to participants and they had to use a 5-point scale with the following alternatives: Completely disagree (1 point), Moderately disagree (2 points), Slightly disagree (3 points), Slightly agree (4 points), Moderately agree (5 points) and Completely agree (6 points), with some items having reverse scoring. The minimum score of this scale is 11 and the maximum score is 66. The NCS was developed by Cacioppo et al. (1984) to measure how much people are attracted to tasks that require intellectual effort. Statements such as “I would prefer complex to simple problems" and "I like to have the responsibility of handling a situation that requires a lot of thinking" are presented to participants and they have to indicate for each of them whether the statement is characteristic of them or not, using the following scale: Extremely uncharacteristic (− 2 points), Somewhat uncharacteristic (− 1 points), Uncertain (0 points), Somewhat characteristic (1 point) and Extremely characteristic (2 points). Reverse scoring was used for some statements. The minimum score in this scale is − 36 and the maximum score is 36.

#### Procedure

After providing consent to participate in this study, participants completed a questionnaire with demographic questions, CRT, AOMTB, and NCS. They were then fitted with a virtual reality headset and controller. Before completing the experiment, participants began by engaging in an interactive VR tutorial to familiarize themselves with the controller functions and to acclimatize to the VR environment. Following this, they completed an instructional walkthrough of the VR-DMX, which was limited to two accessible rooms and two games (Go No Go and Mackworth Clock) for demonstration purposes. No tickets were awarded during the tutorial. A researcher guided participants through the VR-DMX tutorial, explaining how to navigate the arcade, access rooms, game entry and exit procedures and how to track their tickets and time remaining. Participants had the opportunity to play one of the two games during the tutorial before exiting the arcade. The tutorial was designed to acquaint participants with the arcade's operations, ensuring that during the experiment, they could concentrate on accumulating tickets without being distracted by the mechanics of the arcade. Participants then played in the experimental version of the VR-DMX for 30 min. They were instructed to obtain as many tickets as possible.

#### Data analysis plan

Prior to conducting the statistical analysis (detailed below), we conducted data preprocessing on the raw participant files. In a small number of cases, we noticed that the software had output two summary files (for a given participant). For these cases, we chose the most complete data file to include in the analysis. We noticed a minor bug in a handful of participant files such that the last two rows (detailing the arcade end time and the final Total Tickets scored) had been inadvertently duplicated by the software. We corrected this by removing these duplicate rows. We also noticed that a few files had one extra trial row output after 1800 s (i.e., after 30 min had elapsed). We removed any such rows to prevent performance on these trials from influencing the analysis. Lastly, we noticed a minor labeling issue for the trial rows for the ‘Tower of Hanoi—Intermediate’ and ‘Tower of Hanoi—Expert’ games, so we also corrected this. (Scripts for all data cleaning are available on the OSF project under ‘MDMX Study 1 Analysis Bayesian Approach.Rmd’ and MDMX ‘Study 2 Analysis Bayesian Approach.Rmd’.)

The first component of the analysis is to explore the variability of the Total Tickets and the DMX Score measures. The rationale of this exploratory analysis is that any metric that aims to measure how people differ in a trait should have the capacity to detect variability. In order to explore variability and the form of that variability, we obtained a frequency distribution and the following statistics: mean, standard deviation, Skewness, Kurtosis, quartiles and coefficient of variation. The coefficient of variation is the ratio of the standard deviation and the mean of a sample and, if the variable starts at zero, can be interpreted as the relative variability of the sample; in other words, what percentage of the mean the standard deviation is. This measure has been used, for example, in organizational psychology to compare the variability in a measure among different groups of an organization. Given that the coefficient of variation is a scale-free measure because it is standardized it can be used to compare variability in distributions across different measures (see Bedeian et al., 2000).

In order to compare the coefficient of variation of Total Tickets and DMX Score with other variables that have a different than zero lowest score, we subtracted the lowest score from the mean and then we obtained the ratio of the standard deviation and that score. In AOMTB the minimum score is 11, thus the denominator of the ratio was 53.317 (mean)–11 = 42.317. In NCS, the denominator was 6.167 (mean) − (− 36) = 42.167. Once the capacity to detect differences among individuals was established, we investigated how these two metrics correlate with other well-established metrics that measure individual differences in cognitive style: CRT, AOMT, and NCS. This study aims to understanding the characteristics of our two new measures. For this purpose, we conducted a bivariate correlation analysis. As is apparent with other cognitive measures, we also explored the relationship of the measures with age. The statistical analyses were conducted with the statistical software JASP (JASP Team, 2023).

### Results

**Fig. 4:**
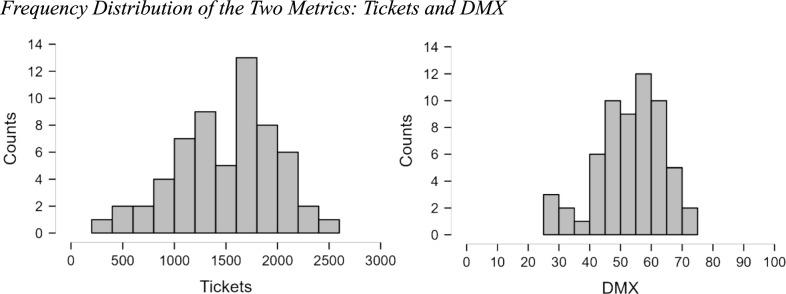
Frequency distribution of the two metrics: tickets and DMX

Raw data, summary data, and analysis scripts are available on the Open Science Framework: https://osf.io/42j7y. The frequency distribution of Total Tickets and DMX Score are presented in Fig. 4. For Tickets, the mean of the distribution was 1509, and the standard deviation is 489. The quartiles of the sample are 1176 (25th percentile), 1591 (median), and 1847 (75th percentile). The skewness (− 0.375) and kurtosis (− 0.260) of the sample suggest a symmetric distribution. The coefficient of variation is 0.324, which indicates that the standard deviation is 32.4% of the mean. Regarding DMX, the mean score in this sample was 53.1, with a standard deviation of 11. The quartiles of the sample are 46.5 (25th percentile), 54.4 (median), and 61.7 (75th percentile). The skewness (− 0.557) and kurtosis of the sample (− 0.002) suggests a symmetric distribution. The coefficient of variation is 0.208, which indicates that the standard deviation is 20.8% of the mean.

The distribution and variability analysis shows that both Total Tickets and DMX Score have a symmetric distribution and a good variability to detect individual differences. The Shapiro–Wilk test suggests that these variables do not significantly deviate from a Normal distribution (Tickets =.984, *p* =.599; DMX =.967, *p* =.109).

Both metrics have a relatively good variability. Here we compare the variability of these two metrics with the variability of other variables in this sample. In this sample, Age (*M* = 22.71; *SD* = 5.48) has a coefficient of variation is 0.241, or 24.1% of the mean. The CRT (*M* = 1.78, *SD* = 1.81) has a coefficient of variation of 1.017 or 101.7% of the mean. Notice that the CRT has excellent variability; however, the distribution is very far from symmetrical, with more than 62% of the sample scoring 0 or 1, and the rest of the sample scoring between 2 and 7 correct answers. The problem with this scale is that the differences among individuals in the lower end of the distribution cannot be detected. The adjusted coefficient of variation (see Methods for details on the applied adjustment) for AOMTB (*M* = 53.3, *SD* = 4.6) was 0.108 or 10.8% of the mean and the adjusted coefficient of variation for the NCS (*M* = 6.17, *SD* = 9.6) was 0.228 or 22.8% of the mean.

We calculated two additional measures of performance in the VR-DMX, to assess whether Total Tickets and DMX Score were indeed measuring unique constructs. We calculated the number of games played by each participant (‘Games’ in Table 1) which can theoretically range from 1 to 21, reflecting the 21 games available in the arcade. The mean number of games played was 13.10 (*SD* = 2.88), with a range of 15 (*Minimum* = 4, *Maximum* = 19). The coefficient of variation was 0.220. We also calculated the number of times each participant switched between games (‘Switches’ in Table 1), to assess how frequently participants were switching in the allocated 30 min. The minimum possible number of switches is 0 and there is no theoretical maximum. The mean number of switches was 18.27 (*SD* = 5.11), with a range of 25 (*Minimum* = 3, *Maximum* = 28). The coefficient of variation was 0.279. The bivariate correlation table is shown in Table 1.

**Table 1:** Bivariate correlations: Study 1

	Tickets	DMX	Games	Switches	CRT	NCS	AOMTB
1. Age	0.316*	− 0.131	− 0.345**	− 0.187	− 0.095	0.193	0.238
2. Tickets		0.593***	0.452***	0.638***	0.323*	0.022	0.019
3. DMX			0.255*	0.458***	0.063	0.091	0.14
4. Games				0.683***	0.079	0.017	0.091
5. Switches					0.052	0.025	− 0.069
6. CRT						0.165	0.333**
7. NCS							0.34**

Pearson *r* scores and Significance correlations *p <.05, **p <.01, ***p <.001 indicate correlations different than 0

This pattern of correlations show that Tickets and DMX have a medium correlation (*r*(58) = 0.593, *p* <.001) indicating that they measure different constructs, albeit people who tend to score high in one of the constructs tend to score high in the other construct. Tickets were also significantly correlated with the Cognitive Reflection Test (*r*(58) = 0.323, *p* = 0.012). Given that the Cognitive Reflection Test correlates with general abilities (e.g., Frederick, 2005), this relationship may be due to the fact that people with a higher general cognitive ability tended to score higher in Tickets. DMX did not correlate with the other cognitive measures. Regarding Age, Tickets significantly and negatively correlated with Age (*r*(58) = − 0.316, *p* = 0.014), showing a decline in performance with age, but DMX did not. The number of switches shows a strong correlation with Tickets (*r*(58) = 0.638, *p* < 0.001) and a moderate correlation with DMX (*r*(58) = 0.458, *p* < 0.001), and these correlations are higher than those between Games and Tickets (*r*(58) = 0.452, *p* < 0.001), and Games and DMX (*r*(58) = 0.255, *p* = 0.049), respectively. These results suggest that switching is a good strategy to gain more tickets as it affords the possibility to explore new games that may be more profitable. Moreover, they suggest that switching to an already played game (which presumable was profitable) is also important as the correlation of total unique games with Tickets and DMX are smaller. The fact that the correlations of Switches with Tickets is stronger than that of Switches with DMX suggests that those who are good at playing games are also good at switching wisely as DMX controls for game expertise.

**Fig. 5:**
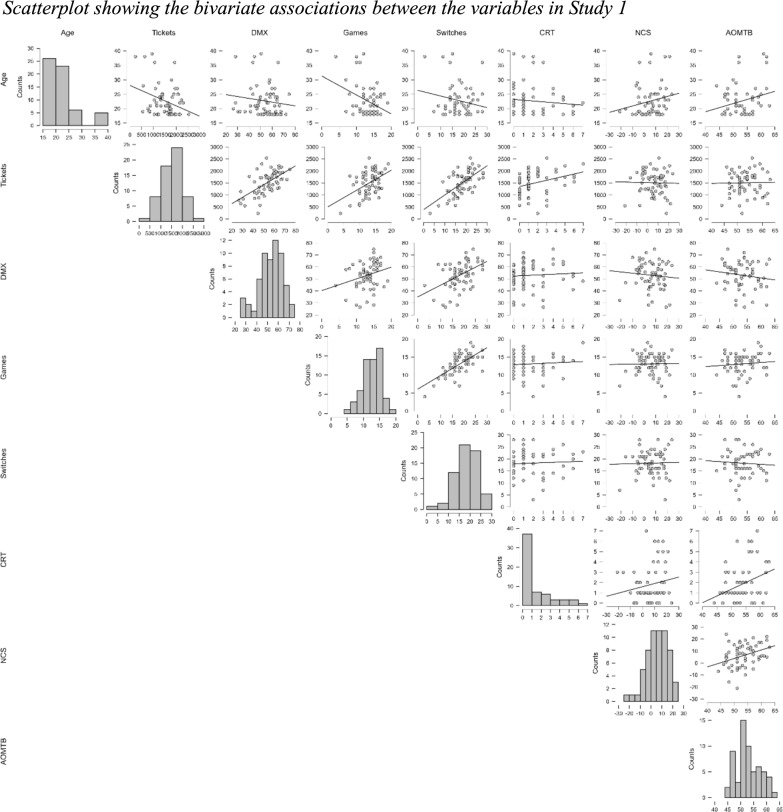
Scatterplot showing the bivariate associations between the variables in Study 1

Scatterplots showing the bivariate associations between the variables in Study 1 can be seen in Fig. 5.

### Discussion

Study 1 shows that both metrics have good distribution properties. They have good variability and their distributions are symmetric. This indicates that both measures are able to detect individual differences in the construct that they are measuring. The overall goal of Study 1 was to evaluate the efficacy of the VR-DMX in identifying individual differences in decision-making ability. Decision-making ability was operationalized using two measures. *Total Tickets* is a measure of each participant’s overall performance within the arcade. *Total Tickets* is purported to be a combined indicator of each participant’s expertise across all the arcade tasks and also their ability to select the ‘best’ (i.e., most profitable) tasks for ticket acquisition. Given the overall goal of the study, we consider it important to mathematically separate these two components. As such, we developed the *DMX Score* measure to capture each participant’s ability to select the ‘best’ tasks for ticket acquisition.

The data from Study 1 indicate that both *Total Tickets* and *DMX Score* are able to elicit sufficient individual differences between participants for the constructs that they are purportedly measuring. The coefficient of variation for *Total Tickets* indicates that the standard deviation for this measure is 32.4% of the mean. Similarly, the coefficient of variation for *DMX Score* indicates that the standard deviation is 20.8% of the mean. Given that the skewness and kurtosis for *Total Tickets* and *DMX Score* are within normal bounds, we can be confident in the psychometric properties of these measures. Importantly, there appear to be no ‘floor’ (i.e., lower bound) or ‘ceiling’ (i.e., upper bound) effects within the sample distribution. That is, participant performance is not artificially constrained by the limits of the measure. Indeed, by the very nature of these measures, there are no limits on the minimum or maximum score that any one participant can achieve—participant scores on each of these measures could theoretically range anywhere from 0 upwards. Reassuringly, the distribution for each of these measures is not bimodal, and there also does not appear to be any ‘clustering’ within the data. This finding suggests that there is little impact of any methodological issues (e.g., multiple interpretations of the instructions) on the distribution of the data. There are also no marked outliers (i.e., no vastly superior or vastly inferior performers), indicating sound psychometric properties of the measures.

The coefficients of variation of 32.4% and 20.8% for the *Total Tickets* and *DMX Score* measures, respectively, fall within the range of other established measures of individual differences. For instance (in this sample), the coefficient of variation for the AOMTB scale is 10.8%, NCS is 22.8%, Age is 24.1%, and the CRT is 101.7%. Discounting the CRT due to its distributional issues, the *Total Tickets* measure appears to have the largest variability while also maintaining a relatively normal distribution. *DMX Score* also appears to be a reasonable measure, with a similar variability to these existing measures. In summary, our results indicate that both *Total Tickets* and *DMX Score* are relatively normally distributed, have sound psychometric properties and their variance is aligned with (or greater than) more established measures of individual differences. As such, this provides us with strong confidence in the ability of these measures to detect individual differences in performance. When considering our eventual use-case for the VR-DMX, we are confident that these measures will be able to detect differences between groups (e.g. emergency responders or pilots, compared to novices) that purportedly differ in decision-making ability (if such differences do indeed exist).

Several correlations were reported to assess the relationships between pairs of variables measured within Study 1. A few correlations were of particular interest. Firstly, *Total Tickets* and *DMX Score* had a significant, positive and medium relationship, such that higher *Total Tickets* scores were moderately associated with higher *DMX Scores*. When considering the basis for how these scores were derived, such a relationship is to be expected. *Total Tickets* was designed to measure each participant’s task expertise, how skilled a person is at completing a specific task, and also their ability to select the ‘best’ tasks for ticket acquisition, tasks that yield a higher number of tickets relative to the time required. In contrast, *DMX Score* was designed to mathematically separate out and measure only the ability to select the ‘best’ tasks for ticket acquisition. As such, given that *DMX Score* measures a constituent component of *Total Tickets*, a correlation between the two measures is to be expected (at least, to the extent that task selection ability accounts for variance in *Total Tickets*). The presence of a medium (as opposed to a strong) relationship also indicates that *Total Tickets* and *DMX Score* are not completely overlapping constructs. That is, they appear to measure (somewhat) different things. This is positive evidence suggesting that the task selection ability that we thought to exist, does exist (at least in a statistical sense). This is also a promising sign for future research. For example, studies testing differences between groups (e.g. emergency responders vs pilots vs novices) would then be able to determine whether purported experts are better decision makers due to their superior task expertise (i.e., how skilled they are at performing individual tasks), task selection ability (i.e., how well they choose the most rewarding or efficient tasks), or both.

The second correlation of interest is the significant correlation between *Total Tickets* and the CRT indicating that those who score high in the VR Arcade game, tend to also score higher in cognitive reflection. On the other hand, *DMX Score* did not correlate with the CRT. This means that whatever the DMX measure is detecting is not related to cognitive reflection. The fact that Tickets and DMX have a medium size correlation (*r*(58) = 0.593) suggests that DMX is measuring something different than Tickets and that different component is not related to cognitive reflection or the general abilities involved in solving the CRT.

The third correlation of interest is the significant, negative and small-medium relationship between Age and *Total Tickets*. In conjunction with this, the lack of a significant relationship between Age and *DMX Score* suggests a unique pattern of results. We interpret and explain these results in the following manner: Firstly, it is well established that cognitive ability declines with age—that is, as people get older, their cognitive ability (as measured on cognitive tasks) worsens. Secondly, we know that our *Total Tickets* measure is constituted by task expertise (i.e., performance on cognitive tasks) and task selection ability. We suggest that Age is negatively correlated with *Total Tickets* because *Total Tickets* is partly constituted by task expertise (i.e., performance on cognitive tasks). Given that older people are likely to perform worse on cognitive tasks, it is likely that they will also have a lower *Total Tickets* score. Essentially, Age correlates with *Total Tickets* because *Total Tickets* is partly a measure of cognitive task performance. Thirdly, the lack of a significant correlation between Age and *DMX Score* makes sense, given that *DMX Score* is not a measure of cognitive task performance (it is only a measure of task selection ability). In summary, these results suggest that Age is correlated with *Total Tickets* because both are associated with cognitive task performance. In contrast, Age is not correlated with *DMX Score*, as *DMX Score* is not constituted by cognitive task performance. This pattern of results is promising as it suggests that our conceptualization of *DMX Score* does in fact mathematically control for the impact of task expertise. That is, this pattern of results suggests that we have successfully disentangled task expertise from task selection ability.

These exploratory age-related interpretations should be considered with caution given the limited age range in our participants, which are heavily skewed toward younger adults. Our findings do not support broad generalizations about age-related decline in cognitive performance or decision-making strategies across the lifespan. Once again, however, this finding is promising for future research as it lends credence to the construct validity of the VR-DMX.

## Study 2

In Study 1, the VR-DMX’s two decision-making measures demonstrated interesting statistical properties in discerning individual differences. To ensure the robustness of these findings, we endeavored to replicate these results in a subsequent study. Moreover, this replication study empowers us to deliver a richer estimation of population parameters by amalgamating the outputs of both Study 1 and Study 2 in a cohesive meta-analysis. The methodology employed mirrors that of Study 1; thus, we detail solely the participants' characteristics in the following Methods section.

### Method

Seventy-eight participants took part in the study. One participant was excluded as they accidentally exited the arcade midway through their session, and one participant was excluded as they withdrew due to illness. In total, valid data was collected from 76 participants (41 female, 33 male, 1 non-binary, 1 deciding) with a mean age of 24.28 (*SD* = 7.39). As in Study 1, participants were university students who took part either for course credit (56 participants) or university-affiliated individuals who received $20 compensation (20 participants). The power target for Study 2 was the same as that specified in Study 1. The materials, data preprocessing and data analysis plan is the same as in Study 1.

### Results

**Fig. 6:**
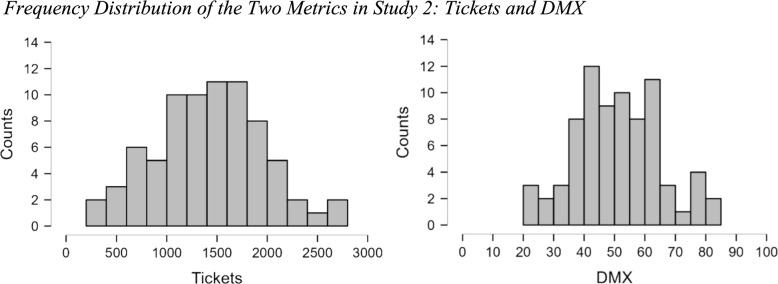
Frequency distribution of the two metrics in study 2: tickets and DMX

Raw data, summary data, and analysis scripts are available on the Open Science Framework: https://osf.io/42j7y. The frequency distribution of *Total Tickets* and *DMX Score* are presented in Fig. 6. For Tickets, the mean of the distribution is 1418, and the standard deviation is 530. The quartiles of the sample are 1090 (25th percentile), 1428 (median), and 1739 (75th percentile). The skewness (0.017) and kurtosis (− 0.245) of the sample suggest a symmetric distribution. The coefficient of variation is 0.374, which indicates that the standard deviation is 37.4% of the mean.

Regarding DMX, the mean score in this sample was 51.1, with a standard deviation of 14. The quartiles of the sample are 40.8 (25th percentile), 50.3 (median), and 60.0 (75th percentile). The skewness (0.136) and kurtosis of the sample (− 0.298) suggests a symmetric distribution. The coefficient of variation is 0.276, which indicates that the standard deviation is 27.6% of the mean.

The results are very similar to those in Study 1, replicating that these two measures are good measures to detect individual differences. As in Study 1, the Shapiro–Wilk test suggests that these variables do not significantly deviate from a Normal distribution (Tickets =.991, *p* =.860; DMX =.983, *p* =.421).

As in Study 1, here we compare the variability of these two metrics with the variability of other variables in this sample. In this sample, Age (*M* = 24.28; *SD* = 7.39) has a coefficient of variation of 0.304, or 30.4% of the mean. The CRT (*M* = 2.51, *SD* = 1.98) has a coefficient of variation of 0.789 or 78.9% of the mean. Notice that compared to Study 1 the CRT has a lower variability, but still a large variability, and also the distribution is more widespread. In Study 1, 62% of the sample scored 0 or 1, whereas in Study 2, 62% of the sample scored 0, 1 or 2, improving the capability to detect differences among people on the lower tail of the scale, albeit still not ideal. The adjusted coefficient of variation (see Methods for details on the applied adjustment) for AOMTB (*M* = 53.4, *SD* = 5.2) was 0.123 or 12.3% of the mean and the adjusted coefficient of variation for the NCS (*M* = 8.79, *SD* = 9.4) was 0.165 or 16.5% of the mean.

Just as in Study 1, we calculated two additional measures of performance in the VR-DMX, to assess whether Total Tickets and DMX Score were indeed measuring unique constructs. The mean number of games played (‘Games’ in Table 2) was 13.54 (*SD* = 2.67), with a range of 13 (*Minimum* = 8, *Maximum* = 21). The coefficient of variation was 0.197. Once again, we also calculated the number of times each participant switched between games (‘Switches’ in Table 2), to assess how frequently participants were switching in the allocated 30 min. The mean number of switches was 18.20 (*SD* = 4.84), with a range of 23 (*Minimum* = 7, *Maximum* = 30). The coefficient of variation was 0.266..

**Table 2:** Bivariate correlations: Study 2

	Tickets	DMX	Games	Switches	CRT	NCS	AOMTB
1. Age	− 0.147	− 0.169	− 0.166	− 0.163	0.005	0.01	0.046
2. Tickets		0.686***	0.318**	0.549***	0.18	0.261*	0.192
3. DMX			0.400***	0.506***	0.021	− 0.066	0.102
4. Games				0.664***	0.055	− 0.167	0.015
5. Switches					− 0.062	− 0.075	− 0.125
6. CRT						0.246*	0.204
7. NCS							0.322**

Pearson *r* scores and Significance correlations * *p* <.05, ** *p* <.01, *** *p* <.001 indicate correlations different than 0

The bivariate correlation table is shown in Table 2.

The correlation between Tickets and DMX is a bit higher than that in Study 1 (*r*(74) = 0.686, *p* <.001), replicating the result in Study 1 that suggests these metrics measure different constructs, albeit people who tend to score high in one of the constructs tend to score high in the other construct. However, unlike Study 1, the correlation between Tickets and the CRT did not reach significance (*r*(74) = 0.180, *p* = 0.120). On the other hand, the correlation between Tickets and NCS (*r*(74) = 0.261, *p* =.023) was significant in Study 2, but not in Study 1. As in Study 1, DMX did not correlate with the other cognitive measures. Regarding Age, unlike Study 1, Tickets did not correlate significantly with Age (*r*(74) = − 0.147, *p* = 0.204).

The number of switches shows a strong correlation with Tickets (*r*(58) = 0.549, *p* < 0.001) and DMX (*r*(58) = 0.506, *p* < 0.001), and these correlations are higher than those between Games and Tickets (*r*(58) = 0.318, *p* = 0.005), and Games and DMX (*r*(58) = 0.400, *p* < 0.001), respectively. This pattern is the same as in Study 1, but not as strong.

**Fig. 7:**
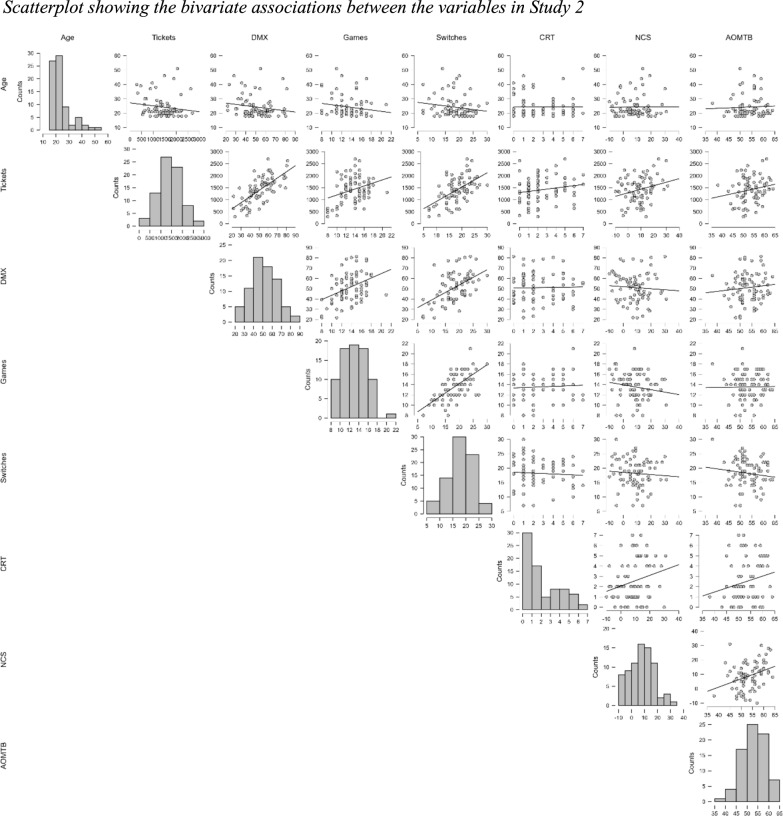
Scatterplot showing the bivariate associations between the variables in Study 2

Scatterplots showing the bivariate associations between the variables in Study 2 can be seen in Fig. 7.

### Discussion

Study 2 replicated the finding in Study 1 that both metrics have good distribution properties in terms of the lack of floor or ceiling effects, and kurtosis and skewness close to zero, suggesting a symmetric distribution also in Study 2. The good variability was replicated in both measures with comparable coefficient of variations, which were slightly higher in Study 2 than in Study 1: in *Total Tickets*, 32.4% in Study 1 and 37.4% in Study 2; in *DMX Score*, 20.8% in Study 1 and 27.6% in Study 2. The significant correlation between *Total Tickets* and *DMX Score* was replicated in Study 2, with the correlation in Study 1 being *r* = 0.593 and that in Study 2 *r* =.686.

In terms of correlations with CRT, we obtained mixed results. As in Study 1 the correlation between DMX and CRT was not significant: *r* =.063 in Study 1, *r* =.021 in Study 2. However, unlike Study 1, in Study 2 we did not replicate the significant correlation between *Total Tickets* and CRT: *r* =.323, *p* =.012 in Study 1, and *r* =.180, *p* =.120 in Study 2. In this case, it will be essential to inspect the results of the meta-analysis, with a more robust estimation of the population correlation parameter. In Study 2, we observed a significant correlation between *Total Tickets* and NCS (*r* =.261, *p* =.023) that we had not observed in Study 1 (*r* = −.022, *p* =.870). We will see in the meta-analysis what the estimate of this correlation is. Finally, the significant negative correlation between Age and *Total Tickets* observed in Study 1 (*r* = −.316, *p* =.014) was not replicated in Study 2 (*r* =.147, *p* =.204). Given the discrepancies between Study 1 and Study 2, we conducted a meta-analysis combining the results of Study 1 and Study 2. The meta-analysis also provides us with a better estimation of the population parameters than the individual separate studies.

## Meta-analysis

We used the effect sizes obtained in Study 1 and Study 2 to obtain a better estimate of the population effects size in a meta-analysis. This meta-analysis with only two studies is inspired by the “New Statistics” concept developed by Cumming (2013), in which the goal of meta-analysis is to provide a more precise estimation of population parameters and to embrace the updating of knowledge based on new information. Notice that Cumming’s approach was incorporated in the American Psychological Association Publication Manual (see Cumming et al., 2012). We were interested in more precisely estimating the population mean of *Total Tickets* and *DMX Score*. We also provided a more precise estimate of the population correlation between *Total Tickets* and *DMX Score* and the population correlation of each of these variables with the cognitive style variables and with Games and Switches. The raw data for the estimation of means were the mean of *Total Tickets* and the mean of *DMX Score* in Study 1 and Study 2, and the corresponding standard errors of the mean. The raw data for the correlation analyses were z scores obtained by transforming the *r* scores into *z* scores, and the corresponding standard error. The raw data or the comparison between means analyses, were *Cohen’s d* scores with their corresponding standard errors. We used the meta-analysis module in JASP to obtain an overall effect size, the corresponding 95% confidence intervals, and *p* values, for each of the effect sizes.

Table 3 shows the results of the meta-analysis for each of the effect sizes. For *Total Tickets* and *DMX Score*, we now have a more precise estimate of their population mean, which is somewhere in between the means in Study 1 and Study 2. The overall effect size for the correlation between the two metrics (Ticket—DMX) is 0.771 with the 95% CI being (0.599, 0.943). Regarding the correlation between Tickets and CRT, which was significant in Study 1 and non-significant in Study 2, the meta-analysis shows a correlation significantly different than 0 (*r* =.249, 95%CI [.077,.420]; *p* =.004). For the correlation between Tickets and NCS, which was non-significant in Study 1 and significant in Study 2, the meta-analysis shows a non-significant correlation (*r* =.129, 95% CI [−.154,.412]; *p* =.372). The meta-analysis confirms the pattern identified regarding Games and Switches, with Switches correlating strongly with Tickets and DMX, and Games correlating moderately with these measures. All the other correlations were non-significant in both studies, and they remained non-significant in the meta-analysis.

**Table 3:** Meta-analysis results for each of the effect sizes

Effect size	Study 1		Study 2		Overall				
	Effect size	SE	Effect size	SE	Effect size	95%CI LB	95%CI UB	*z*	*p*
Tickets (mean)	1508.7	63.2	1418.8	60.8	1462.1	1374.2	1550.1	32.58	<.001
DMX (mean)	53.1	1.4	51.1	1.6	52.2	50.1	54.3	48.84	<.001
Tickets-age	− 0.328	0.132	− 0.149	0.117	− 0.228	− 0.402	− 0.054	− 2.57	0.1
Tickets-DMX	0.682	0.132	0.841	0.117	0.771	0.599	0.943	8.81	<.001
Tickets-games	0.487	0.132	0.329	0.117	0.399	0.227	0.570	4.55	<.001
Tickets-switches	0.755	0.132	0.616	0.117	0.677	0.506	0.849	7.73	<.001
Tickets-CRT	0.334	0.132	0.182	0.117	0.249	0.077	0.420	2.84	0.004
Tickets-AOMBT	0.019	0.132	0.194	0.117	0.117	− 0.055	0.289	1.34	0.181
Tickets-NCS	− 0.022	0.132	0.267	0.117	0.129	− 0.154	0.412	0.89	0.372
DMX-Age	− 0.132	0.132	− 0.171	0.117	− 0.154	− 0.325	0.018	− 1.76	0.079
DMX-games	0.261	0.132	0.423	0.117	0.352	0.180	0.523	4.02	<.001
DMX-switches	0.495	0.132	0.557	0.117	0.530	0.358	0.701	6.05	<.001
DMX-CRT	0.063	0.132	0.021	0.117	0.039	− 0.132	0.211	0.45	0.652
DMX-AOMBT	− 0.141	0.132	0.102	0.117	− 0.012	− 0.249	0.226	− 0.10	0.922
DMX-NCS	− 0.091	0.132	− 0.066	0.117	− 0.077	− 0.249	0.095	− 0.88	0.379

The effect sizes are explained in the text. SE stands for standard error of the corresponding effect size, 95CI LB is the lower bound of the 95% confidence interval and 95CI UB is the upper bound of the 95% confidence interval. In all cases, the *p* values were obtained by comparing the obtained *z* score with the null hypothesis of a *z* score of 0

## General discussion

The goal of this study was to develop a measure of decision-making expertise, inspired by safety–critical professions, that neither measures a very general cognitive ability nor is overly specific. We aimed, therefore, to develop an environment that assesses an ability applicable across various situations, and that bears some resemblance to real-world tasks while remaining broadly applicable beyond in situ use. With these considerations in mind, we developed a virtual reality arcade game scenario, rather than the traditional desktop-based decision-making tasks, to increase ecological validity.

We examined the distributional properties of the two measures and found that both effectively detect individual differences. The distribution of participant scores in both measures across both studies was symmetrical, exhibiting neither a floor nor a ceiling effect and displaying a broad range of values. This indicates that both measures can detect differences across all levels of expertise. For example, since the distributions are symmetrical and lack a floor effect, differences among individuals with low scores can be detected. Additionally, the symmetry and absence of a ceiling effect enable the detection of differences among individuals with high expertise. The two measures are moderately correlated, indicating that they capture a shared component while also assessing distinct aspects of decision making. This distinction was further reflected in their varying correlations with the CRT. Total Tickets, which primarily assesses participants’ expertise in the games, correlates with general cognitive abilities like cognitive reflection. In contrast, DMX, which measures decision-making expertise in resource allocation, did not correlate with the Cognitive Reflection Test.

During the validation of the VR-DMX, we became aware of a similar approach (see Wells et al., 2021). They developed COGMISSION, a battery of cognitive tasks designed in the style of a role-playing first-person shooter video game to measure cognitive control abilities, including working memory, response inhibition, attention control, multitasking, task switching, and the use of prior information. COGMISSION, developed by Wells et al., gamified 10 cognitive control tasks, including the n-back task and the stop-signal task, within a first-person shooter video game. The goal was to maintain engagement during the extended testing required to reliably measure performance differences on these tasks. However, COGMISSION is limited to the construct of cognitive control, whereas our focus is on decision-making expertise using virtual reality.

To conclude, this special issue of aimed to explore cognitive processes within military settings, emphasizing use-inspired basic research and to help bridge the gap between basic research and real-world applications. The VR-DMX represents a direct response to this call by developing a measurement approach inspired by the cognitive demands of military contexts while contributing to basic understanding of decision-making processes that could span multiple applied domains. We designed VR-DMX specifically to tap into domain-general cognitive processes that we hypothesize are important for effective performance in military contexts, such as exploration–exploitation decision making under uncertainty, strategic resource allocation under time pressure, dynamic learning from limited feedback, and metacognitive monitoring of performance effectiveness.

Our two studies represent the initial step in the validation process of VR-DMX. In the future, this environment could be administered to experts who are expected to excel in realistic decision-making scenarios, such as pilots, firefighters, police officers, and submariners. It has the potential to serve as an instrument for investigating the cognitive processes underlying expert decision making. It could be adapted into a training tool for novices in decision making or used to assess personnel decision-making capacity for selection and predictive purposes. While more validation and performance data are needed before it can be effectively translated for use in practice, we think that it contributes to basic mechanistic and theoretical models of decision making while also holding future potential for enhancing training, performance, and operational effectiveness. By creating a task environment and novel measure that bridges laboratory-based cognitive assessment with military-relevant decision-making demands, we hope to have contributed to the special issue's broader goal of advancing cognitive science through military-inspired research that addresses real-world challenges.

## Supplementary Information

Additional file 1.

## Data Availability

Raw data, summary data, and analysis scripts are available on the Open Science Framework: https://osf.io/42j7y
