# Supplementary material for: Detecting expertise in decision making under pressure: a virtual reality assessment environment and empirical evaluation
Source: Cogn Res Princ Implic. 2026 Jan 8;11:6. doi: 10.1186/s41235-025-00695-6 (PMC12783459; doi:10.1186/s41235-025-00695-6)
Supplement: Supplementary file 1 — Additional file 1. [file 41235_2025_695_MOESM1_ESM.docx]

# Description of Tasks in the VR-DMX

## Corsi block-tapping task (Corsi, 1972)

In Corsi, participants must remember and reproduce sequences. Participants watch the spheres, arranged in a three-by-three grid, light up in a random sequence and then reproduce the sequence in the same order. The sequences start with two spheres and gradually increase in length up to nine spheres. Participants are given eight to ten trials at each sequence length. There are two versions of the Corsi task in the VR-DMX, a standard and expert version. The standard version begins with a sequence of two, while the expert version begins with a sequence of six.

## Go No-Go (Donders, 1868)

Participants are asked to respond to a series of green "Go" buttons and red "No-Go" buttons. Participants are instructed to click the green "Go" button when it appears, while refraining from responding to the red "No-Go" button. The task is comprised of various trials, with a 50-50 chance of either the "Go" or "No-Go" button being presented throughout the task. The primary objective is to test participants' response inhibition abilities. The VR-DMX has two versions of the Go No-Go task, a standard version and Go No-Go 100. Go No-Go 100 requires participants to get 100% of their responses correct to win tickets.

## The Iowa Gambling Task (Bechara et al., 1994)

Participants are given a $2000 credit and instructed to maximize their profit by selecting cards from four card decks. Participants are told to choose cards from the decks and win as much money as possible. Each card selection results in either a monetary gain or loss, two of the card decks (the "risky" decks) have high gains but also high losses, while the other two card decks (the "safe" decks) have lower gains but also lower losses. Participants learn through trial and error which card decks are more advantageous and which are riskier, typically adjusting their strategy accordingly. There are no additional versions of this game in the VR-DMX.

## Mackworth Clock (Time Defender) (Mackworth, 1948)

Participants are presented with a clock face and are asked to monitor the clock for any changes in the position of the minute hand. The minute hand of the clock moves at a fixed rate, but occasionally it skips a position, making a greater jump forward than usual. Players are instructed to press a button labelled “JUMP” as soon as they detect a jump in the position of the minute hand, and the reaction time and accuracy of their responses are recorded. The VR-DMX has three versions of the Mackworth Clock task, a standard, extreme and ultimate version. The standard version has a 50 percent chance that a greater jump will occur. The extreme version has a 40 percent chance that a greater jump will occur. Lastly, the extreme version has a 30 percent chance that a greater jump will occur. The decreased chance of a jump means participants need to stay vigilant for longer to notice and respond to a jump.

## Mixed Hybrid Search (Target Termination)

Participants are presented with a search area, that contains a variety of objects, some of which are target objects that they need to identify. Participants shown two specific objects and given one object category that they will need to search for among other objects in the search area. Participants target objects appear randomly in the search area. There are three versions of the mixed hybrid search task in the VR-DMX, a standard, extreme and a Time Crunch version. In the standard version of Mixed Hybrid Search participants have 20 seconds per trial to search for target objects. In the extreme version, participants have 10 seconds of search time and 5 seconds of search time the Time Crunch version.

## Posner Letter Match Task (Posner et al., 1969)

Participants are presented with pairs of letters and asked to indicate whether the letters are the same or different according to a specific rule. The letters can be presented in the same case (e.g., A-A or B-b) or different cases (e.g., A-a or B-b). The VR-DMX has three versions of the Posner Letter Match Task, Name, Physical Identity and a Consonants and Vowels version. In the Name version, participants compare and decide whether pairs of letters have the same name, (e.g., “a A”) or different names (e.g., “C E”). In the Physical Identity version, participants are presented with pairs of letters that are either physically identical (e.g., "A A" or "b b") or different ("A a" or "B A"). The Consonants and Vowels version of the task involves participants identifying whether pairs of letters are either two consonants (e.g., "A A" or "B b") or two vowels (e.g., A B). Participants must indicate whether the two letters are the same (both consonants or vowels), or different. In all versions of the Posner Letter Match Task participants must press a button indicating whether the pairs of letters are either the same or different according to the specific rule of each version.

## Mental Rotation (Shape Shifter) (Shepard & Metzler, 1971)

In this task, pairs of 3D shapes, each rotated to different degrees, are presented to participants. Participants must decide whether the two shapes are the same or different. Mental rotation typically involves multiple of trials, with the rotation angle of the shapes varying across trials to create different levels of difficulty. The mental rotation task assesses individuals’ ability to mentally rotate two-dimensional or three-dimensional objects. There are two versions of the mental rotation task in the VR-DMX, a standard and topsy turvy version, where one of the shapes is inverted.

## Stroop (Stroop, 1935)

Participants are presented with colour names (e.g., red, green, blue, yellow) printed in congruent ink colours (e.g., the word “red” is printed in red) and incongruent ink colours (e.g., the word “red” is printed in blue) are presented to participants. Participants are asked to name the ink colour as quickly as possible, ignoring the word. Participants must selectively attend to a particular piece of information while suppressing distracting information. There are two versions of the Stroop task in the VR-DMX, a standard and an all-incongruent version.

## Tower of Hanoi (Lucas, 1884)

Participants are presented with three pegs and a series of discs of different sizes that are stacked on one peg. The goal of the game is to move all the discs from the left starting peg to the destination peg, the peg on the far right, using the middle peg as a temporary holding place. Participants can only move one disc at a time, and they must always move the top disc from one peg to another. Additionally, larger discs cannot be placed on top of smaller discs, so players must plan their moves carefully to avoid getting stuck. The greater the number the of discs the participant must move, the more difficult the task becomes. There are 3 versions of Tower of Hanoi in the VR-DMX, a standard, intermediate and expert version. The standard version of Tower of Hanoi has three discs, intermediate as four and expert has five.

## References

Bechara, A., Damasio, A. R., Damasio, H., & Anderson, S. W. (1994). Insensitivity to future consequences following damage to human prefrontal cortex. *Cognition, 50*(1–3), 7–15.

Corsi, P. M. (1972). Human memory and the medial temporal region of the brain. Dissertation Abstracts International, 34 (02), 891B. (University Microfilms No. AAI05–77717).

Donders, F. C. (1969). On the speed of mental processes. *Acta Psychologica, 30*, 412–431. (Original work published 1868)

Mackworth, N. H. (1948). Mackworth Clock Test. *The Quarterly Journal of Experimental Psychology, 1*, 6-21.

N. Claus (pseudonym for Edouard Lucas) (1884). La Tour d’Hanoi: Jeu de Calcul. *Science et Nature, 1(8)*, 127–128.

Posner, M. I., Boies, S. J., Eichelman, W. H., & Taylor, R. L. (1969). Retention of visual and name codes of single letters. *Journal of Experimental Psychology, 79*(1, Pt.2), 1–16.

[https://doi.org/10.1037/h0026947](https://psycnet.apa.org/doi/10.1037/h0026947)

Shepard, R. N., & Metzler, J. (1971). Mental rotation of three-dimensional objects. *Science*, *171*(3972), 701-703.

Stroop, J. R. (1935). Studies of inference in serial verbal reactions. *Psychological Monographs, 50*, 38-48.

# Computational Approach - Total Tickets and DMX Score

This section describes the computational approach taken to calculate the two measures of expertise reported in the paper: *Total Tickets* and *DMX Score*. As mentioned, Total Tickets is a measure of overall expertise (i.e. it is influenced by game performance and game selection ability), whilst DMX Score is a measure of decision-making expertise (i.e. it is a measure of the quality of choices made and is influenced only by game selection ability). Whilst designing our computational approach, we developed two variant measures of decision-making expertise (i.e. two ‘types’ of DMX score) — and we explain both below — however, readers should note that only the ‘Updated Choice Value’ measure is reported in the paper (as ‘DMX Score’), given that we found the two measures were highly correlated.

Measure 1: Total Tickets

This measure quantifies participant performance (i.e. overall expertise) in the VR-DMX. Overall expertise is measured by the number of tickets earned by a player in the arcade within the limited time provided (i.e. 30 minutes). Better performance is indicated by a greater number of tickets earned and poorer performance is indicated by fewer tickets earned. Participants earn tickets based on their performance in each game that they play. Therefore, Total Tickets can be calculated by summing the tickets earned across all plays.

$$Total Tickets= \sum_{i=1}^{N} {Tickets Earned}_{i}$$

Here, *i* indexes the play number and *N* is the total number of plays made. Note that a ‘play’ refers to each time a participant enters a game. Not all games in the arcade will have the same number of plays and some games might have 0 plays. The total number of plays made (*N*) will vary between participants, as some participants may spend less time on each play (and therefore complete many plays in the limited time provided – high *N*), whilst others will spend more time on each play (and therefore complete less plays in the limited time provided – low *N*).

As an example, consider Participant A who played the following games, in this order:
Play 1: Stroop – 50 tickets 🡪 Play 2: Go No-Go – 70 Tickets 🡪 Play 3: Go No-Go – 20 tickets 🡪 Play 4: Posner Letter Match – 60 tickets 🡪… Play *N*: Corsi – 40 tickets.
The Total Tickets earned for Participant A would be given by:

$$Total Tickets= {Tickets Earned}_{1}+{Tickets Earned}_{2}+{Tickets Earned}_{3}+{Tickets Earned}_{4}+\ldots{Tickets Earned}_{N}$$

$$\therefore{Total Tickets}_{Participant A}= 50+70+20+60+\ldots40$$

It is important to note that Total Tickets earned is a function of i) task performance and ii) task selection. A participant with expertise in a task will naturally perform better and earn more tickets than a participant without expertise. For example, a participant with greater visual working memory will perform better in Corsi, compared to a participant with poorer visual working memory.
Alternatively, a participant that has better task selection ability will earn more tickets than a participant with poorer task selection ability. For example, a participant that chooses more profitable tasks to play will also naturally earn more tickets than a participant who chooses less profitable tasks to play. Note that ‘profitability’ itself is likely a function of i) task characteristics (set by the experimenter) and ii) participant expertise with the task. Regardless, a participant’s ability to assess ‘profitability’ is what we are interested in and we outline how we mathematically control for task expertise in the ‘Development of Measures 2 and 3’ section below.

Given that Total Tickets is influenced by task performance and task selection, this measure fails to tell us whether a participant’s overall level of performance is attributable to their expertise with the task or to their task selection ability. In fact, it is entirely possible for two participants to have the same level of overall expertise (e.g. Total Tickets_Participant A_ = Total Tickets_Participant B_ = 1000), but have different levels of task performance and task selection. For example, Participant A may perform poorly on the tasks but may compensate for this by choosing more profitable tasks. In contrast, Participant B may perform very well on the tasks but may be hampered due to their selection of less profitable tasks. As such, although we expect that the Total Tickets measure will still predict important individual differences between people, it is ‘contaminated’ by the dual influences of task performance and task selection. We addressed this by developing Measures 2 and 3 which are described below.

Development of Measures 2 and 3

To mathematically distinguish between task performance and task selection (i.e. decision-making expertise), we needed to translate our conceptual definition of decision-making expertise into an operational one. That is, what does it mean for someone to make a ‘high quality decision’ in the VR-DMX?

In order to answer this, we reflected on what ‘quality decisions’ look like in safety-critical professions and domains of expertise. For instance, consider a submariner responding to a time-sensitive emergency where they need to decide between high reward tasks (e.g. extinguishing a fire) or low reward tasks (e.g. checking weather conditions). Supposedly, this type of decision making requires an understanding of the ‘reward’ gained for completing different tasks and an understanding of the time required for each of these tasks. Additionally, ‘reward’ may be dynamically changing (i.e. checking weather conditions may be ‘low reward’ during an on-board fire emergency, but may be ‘high reward’ during an impending storm) and is aligned to some overall outcome (e.g. safety – extinguishing a fire increases safety during a fire emergency, but checking the weather probably would not).

As such, our conceptualization of decision-making expertise is one that reflects this ability to discriminate between high and low reward tasks. A person that has decision-making expertise will more frequently select high reward tasks, whilst a person without decision-making expertise will select low reward tasks. Given that the overall outcome of the VR-DMX scenario is to earn as many tickets as possible within the limited time available, ‘reward’ is operationalized as the number of tickets earned per second by a participant (earlier referred to as ‘profitability’ of a task). As such, a participant that has decision-making expertise will more frequently select highly profitable games (i.e. those that provide many points per second) whilst a participant without decision-making expertise will select games that are low in profitability (i.e. those don’t provide many points per second). Measure 2: Choice Value and Measure 3: Updated Choice Value are designed to capture how well participants can choose tasks that are highly profitable. Operationally, this is quantified by summing a transformed profitability value of every decision (called the choice value) made by participants within the VR-DMX. The Measure 2: Choice Value and Measure 3: Updated Choice Value sections below describe the calculation of each measure.

Measure 2: Choice Value

For the purposes of this analysis, profitability value is interchangeably referred to as the ‘tickets per second’ earned by completing a particular task. To evaluate a participant’s decision-making expertise, our approach is to sum the choice values of every decision made by a participant. At every point in time that they are in the arcade, participants are free to either remain in the game that they are currently playing, or they can choose to move to one of the other 20 games available. As such, we consider participants to be making decisions at every point in time that they are in the arcade. Operationally (and practically), we define a point in time as a 1-second time period. Therefore, participants are deciding to remain or change game at every second that they are in the arcade. The total choice value (i.e. the sum of choice values across time points) is given by:

$$Total Choice Value= \sum_{t=1}^{1800} {Choice Value}_{t}$$

*t* indexes the time point (or temporal bin). Assuming that participants spend 30 minutes (i.e. 1800 seconds) in the arcade, *t* has a maximum value of 1800. Essentially, we are summing the value of every decision made. A participant that has a greater total choice value is deemed to be a better decision-maker compared to a participant with a lower total choice value.

The next step is to calculate the choice value of each of the decisions made. Notably, we cannot use the raw profitability (i.e. tickets per second) for each game, as this value is still ‘contaminated’ by the influence of task performance. Below, we outline the series of transformations used to control for task performance and isolate decision-making performance.

The number of tickets gained in a particular game is given by:

$${Total Tickets}_{g}= \left\{ \begin{aligned} \sum_{i=1}^{N_{g}} {Tickets Earned}_{gi} \\ 0 \end{aligned} \right. \begin{matrix} for N_{g}>0 \\ for N_{g}=0 \end{matrix}$$

Here, *g* indexes the game (i.e. 1 = Tower of Hanoi, 2: Mackworth Clock, 3…) and can range from 1 to 21, *i* indexes the play number and *N_g_* is the total number of plays made for game *g*. *Tickets Earned_gi_* is the number of tickets earned for play *i* of game *g*. When a game has no plays, *Total Tickets*_g_ is 0.

The number of seconds spent playing a particular game is given by:

$${Total Seconds}_{g}= \left\{ \begin{aligned} \sum_{i=1}^{N_{g}} {Time Spent}_{gi} \\ 0 \end{aligned} \right. \begin{matrix} for N_{g}>0 \\ for N_{g}=0 \end{matrix}$$

*Time Spent_gi_* is the number of seconds for play *i* of game *g*. When a game has no plays, *Total Seconds*_g_ is 0.

The next step is to calculate the profitability (i.e. tickets per second) for each game. This is calculated by dividing the number of tickets gained in each game by the number of seconds spent playing each game, respectively.

$${Profitability}_{g}= \frac{{Total Tickets}_{g}}{{Total Seconds}_{g}}= \left\{ \begin{aligned} \frac{\sum_{i=1}^{N_{g}} {Tickets Earned}_{gi}}{\sum_{i=1}^{N_{g}} {Time Spent}_{gi}} \\ 0 \end{aligned} \right. \begin{matrix} for N_{g}>0 \\ for N_{g}=0 \end{matrix}$$

*Profitability* gives us the first indication of the value of each game to the participant. However, it is still impacted by the influence of task performance. Participants that are more skillful at the games will naturally have a higher total profitability when summing across all games, compared to participants that are less skillful. To help us control for expertise, we can normalize these profitability values by expressing them as a proportion of the total profitability across games (*Norm Profitability*):

$${Norm Profitability}_{g}=\frac{{Profitability}_{g}}{\sum_{g=1}^{21} {Profitability}_{g}}=\frac{\left[ \frac{{Total Tickets}_{g}}{{Total Seconds}_{g}} \right]}{\sum_{g=1}^{21} \left[ \frac{{Total Tickets}_{g}}{{Total Seconds}_{g}} \right]}$$

$${Norm Profitability}_{g}=\left\{ \begin{aligned} \frac{\left[ \frac{\sum_{i=1}^{N_{g}} {Tickets Earned}_{gi}}{\sum_{i=1}^{N_{g}} {Time Spent}_{gi}} \right]}{\left[ \sum_{g=1}^{21} \left[ \frac{\sum_{i=1}^{N_{g}} {Tickets Earned}_{gi}}{\sum_{i=1}^{N_{g}} {Time Spent}_{gi}} \right] \right]} \\ 0 \end{aligned} \right. \begin{matrix} for N_{g}>0 \\ for N_{g}=0 \end{matrix}$$

*Norm* *Profitability_g_* provides us with a normalised measure of the value of each game to the participant. Additionally, in all participants:

$$\sum_{g=1}^{21} {Norm Profitability}_{g}=1$$

This means that the sum of all *Norm Profitability* values is the same for each participant. These values are no longer influenced by task performance. Rather, the *Norm Profitability_g_* of a particular game is a measure of the proportion of the overall profitability that game *g* accounts for. For example, if *g* = 1 (i.e. Game: Tower of Hanoi) and
*Norm Profitability*_1_ = 0.11, then choosing to play Tower of Hanoi would award a value of 0.11. Given that the average *Norm Profitability* value = 0.04 (i.e. 1/21), it would appear that choosing Tower of Hanoi is a relatively good decision.

However, it is important to note that *Norm Profitability* values will be heavily skewed by very large or very small values. If a certain game has a very large *Norm Profitability*, the relative value assigned to other games would be very small. In this case, a participant would find it difficult to make high value decisions, unless they only chose the game with the very large *Norm Profitability*. To reduce the impact of this, we suggest also calculating the rank order of each game (based on the *Norm Profitability* values), and then factoring these ranks into the final choice value calculation.

$${Norm Rank}_{g}=\frac{{Rank}_{g}}{\sum_{g=1}^{21} {Rank}_{g}}$$

*Rank_g_* is the rank order of game *g*, according to its *Norm Profitability_g_* value for that participant. The game with the lowest *Norm Profitability* is assigned rank = 1, and each game is assigned a rank up to rank = 21. *Norm Rank_g_* is a normalised ranking. When summed across all games, *Norm Rank_g_* = 1.

We can now calculate the Choice Value for each game by averaging *Norm Profitability_g_* and *Norm Rank_g_*.

$${Choice Value}_{g}=\frac{{Norm Profitability}_{g}+{Norm Rank}_{g}}{2}$$

$${Choice Value}_{g}=\frac{\left( \frac{{Profitability}_{g}}{\sum_{g=1}^{21} {Profitability}_{g}} \right)+\left( \frac{{Rank}_{g}}{\sum_{g=1}^{21} {Rank}_{g}} \right)}{2}$$

*Choice Value_g_* provides us with a normalised, rank-adjusted measure of the value of choosing game *g*. A higher value indicates that choosing game *g* is a better decision. We now need to take each participant’s decisions and assign the corresponding Choice Value for that decision. For example, if a participant chose Mackworth Clock (i.e. *g* = 3) at *t* = 450, they would be assigned *Choice Value_3_* for that decision. We repeat this process for all 1800 decisions made by each participant. We then take the sum of these values to calculate the *Total Choice Value*:

$$Total Choice Value= \sum_{t=1}^{1800} {Choice Value}_{t}$$

*Total Choice Value* is Measure 2. If we let *Choice Value_td_* represent the *Choice Value* of the participant’s decision *d* at time *t*, we have:

$$Total Choice Value= \sum_{t=1}^{1800} {Choice Value}_{td}$$

$$Total Choice Value= \sum_{t=1}^{1800} \left[ \frac{{Norm Profitability}_{d}+{Norm Rank}_{d}}{2} \right]$$

$$Total Choice Value= \sum_{t=1}^{1800} \left[ \left( \left( \frac{\left[ \frac{{Total Tickets}_{d}}{{Total Seconds}_{d}} \right]}{\sum_{g=1}^{21} \left[ \frac{{Total Tickets}_{g}}{{Total Seconds}_{g}} \right]} \right)+\left( \frac{{Rank}_{d}}{\sum_{g=1}^{21} {Rank}_{g}} \right) \right)/2 \right]$$

$$Total Choice Value= \sum_{t=1}^{1800} \left[ \left( \left( \left\{ \begin{aligned} \frac{\left[ \frac{\sum_{i=1}^{N_{d}} {Tickets Earned}_{di}}{\sum_{i=1}^{N_{d}} {Time Spent}_{di}} \right]}{\left[ \sum_{g=1}^{21} \left[ \frac{\sum_{i=1}^{N_{g}} {Tickets Earned}_{gi}}{\sum_{i=1}^{N_{g}} {Time Spent}_{gi}} \right] \right]} \\ 0 \end{aligned} \right. \begin{matrix} for N_{d}>0 \\ for N_{d}=0 \end{matrix} \right)+\left( \frac{{Rank}_{d}}{\sum_{g=1}^{21} {Rank}_{g}} \right) \right)/2 \right]$$

This is the *Total Choice Value* expressed in expanded form. Measure 2 has numerous advantages. Particularly, it can control for task performance and isolate the influence of decision-making expertise – the very construct that we purport to measure. However, a major disadvantage of this measure is that all variables are calculated ‘retrospectively’. By this, we mean that the *Choice Value_g_* for game *g* is calculated using performance data from the participant’s entire time in the arcade (i.e. from *t* = 0 to *t* = 1800), using *Total Tickets* and *Time Spent* values across all plays of game *g*. The issue with this becomes more apparent when we consider a participant’s first few minutes in the arcade. For instance, when a participant chooses to play Stroop for the first time (e.g. at *t* = 40) they will be assigned a choice value for that decision. However, from the participant’s perspective, they did not have any information about that game at that point in time. Essentially, we are assessing this participant on a decision made when only 40 seconds worth of information was available to them, even though a ‘good’ decision might require many more seconds worth of information. The impact of this issue fades as *t* tends towards 1800, and as the participant gains more information about the games. We aim to further address this issue using Measure 3: Updated Choice Value.

Measure 3: Updated Choice Value

This measure has the same rationale as Measure 2. We are still assessing participant decisions at each time point (i.e. from *t* = 1 to *t* = 1800), however we are assessing these decisions based on the information available to the participant at each time point. For example, when we assess a participant’s decision at *t* = 650, we will calculate Choice Values for each game using the game data from *t* = 0 to *t* = 650. We will then assign a Choice Value depending on the decision made at this time point. This process is repeated at every time point from *t* = 1 to *t* = 1800. Essentially, the choice value for each game changes over time, rather than being a fixed value.

$$Updated Total Choice Value= \sum_{t=1}^{1800} {Updated Choice Value}_{td}$$

*Updated Choice Value_td_* refers to the Choice Value of the participant’s decision *d* at time *t*, using the game data available up to time *t* only. In essence, this approach computes the exact same calculation used for *Total Choice Value* for Measure 2, except this calculation is done repeatedly for many different time intervals.

$$Updated Total Choice Value= \sum_{t=1}^{1} {Choice Value}_{td}+\sum_{t=1}^{2} {Choice Value}_{td}+\sum_{t=1}^{3} {Choice Value}_{td}+\sum_{t=1}^{4} {Choice Value}_{td}+\sum_{t=1}^{5} {Choice Value}_{td}+\ldots\sum_{t=1}^{1800} {Choice Value}_{td}$$

The first expression in this series calculates the *Choice Value* of the participant’s decision *d* at time *t*, using the game data available up to *t* = 1 only. The next expression increments this by 1, and calculates the *Choice Value* of the participant’s decision *d* at time *t*, using the game data up to *t* = 2 only. This pattern continues in this manner. Finally, summing all of the expressions provides the *Updated Total Choice Value*. The main advantage of this measure is that it better reflects the information available to each participant at each point in time – philosophically, it feels ‘fairer’ to assess performance on the basis of information that is actually shown to participants. A potential disadvantage of this measure is that it requires greater computational power and/or time to calculate. Our analyses on the pilot data takes about 3 minutes per participant to complete, on a moderately powerful computer. As such, we don’t foresee this to be a major issue going forward. It is worthwhile to also mention that analysis of our pilot testing data indicates that Measure 2 and Measure 3 are highly correlated. This is positive news in that it confirms our intuitions that both approaches are practically very similar (though philosophically slightly different). In fact, taken to the extreme, we can expect the two measures to eventually converge. Given infinite time in the arcade for a hypothetical participant, calculating the *Choice Values_g_* using Measure 2 would be nearly identical to the *Updated Choice Values_g_* using Measure 3.
